# miRNAs Involved in Esophageal Carcinogenesis and miRNA-Related Therapeutic Perspectives in Esophageal Carcinoma

**DOI:** 10.3390/ijms22073640

**Published:** 2021-03-31

**Authors:** Giovanni Zarrilli, Francesca Galuppini, Valentina Angerilli, Giada Munari, Marianna Sabbadin, Vanni Lazzarin, Lorenzo Nicolè, Rachele Biancotti, Matteo Fassan

**Affiliations:** 1 Surgical Pathology & Cytopathology Unit, Department of Medicine (DIMED), University of Padua, 35121 Padua, Italy; giovanni.zarrilli@gmail.com (G.Z.); francesca.galuppini@unipd.it (F.G.); valentina.angerilli@gmail.com (V.A.); mariannasabbadin310@gmail.com (M.S.); vanni.lazzarin@unipd.it (V.L.); lorenzo.nic86@gmail.com (L.N.); racheb@hotmail.it (R.B.); 2 Veneto Institute of Oncology-IOV-IRCCS, 35128 Padua, Italy; giada.munari@gmail.com

**Keywords:** microRNAs, esophageal cancer, carcinogenetic cascade, predictive biomarker, targeted therapy

## Abstract

MicroRNAs (miRNAs) are small non-coding RNAs that play a pivotal role in many aspects of cell biology, including cancer development. Within esophageal cancer, miRNAs have been proved to be involved in all phases of carcinogenesis, from initiation to metastatic spread. Several miRNAs have been found to be dysregulated in esophageal premalignant lesions, namely Barrett’s esophagus, Barrett’s dysplasia, and squamous dysplasia. Furthermore, numerous studies have investigated the alteration in the expression levels of many oncomiRNAs and tumor suppressor miRNAs in esophageal squamous cell carcinoma and esophageal adenocarcinoma, thus proving how miRNAs are able modulate crucial regulatory pathways of cancer development. Considering these findings, miRNAs may have a role not only as a diagnostic and prognostic tool, but also as predictive biomarker of response to anti-cancer therapies and as potential therapeutic targets. This review aims to summarize several studies on the matter, focusing on the possible diagnostic–therapeutic implications.

## 1. Introduction

Esophageal carcinoma (EC) is the 7th most frequent malignancy worldwide, with 570,000 new cases (Male/Female ratio: 2–2.5/1) and 508,500 deaths in 2018 [1].

The vast majority (95%) of esophageal carcinomas are adenocarcinomas (EACs) or squamous cell carcinomas (ESCC). These two entities have different pathogenesis and localization, and partially different risk factors.

EAC arises almost exclusively in the lower third of the organ and is associated with gastroesophageal reflux disease, cigarette smoking, and obesity. Barrett’s esophagus (BE) can be defined as a metaplastic transformation of the esophageal squamous epithelium into an intestinal-type epithelium and is a preneoplastic lesion that can progress into EAC. The definition of BE is controversial; in fact, many authors place any type of columnar metaplasia of the esophagus into the BE category [2,3]. For this review, we are defining BE only as the intestinal-type metaplasia of the esophageal epithelium. BE is the result of a chronic damage upon the squamous esophageal epithelium, whose staminal cells are reconditioned to mature in a columnar phenotype. The metaplasia can progress to dysplasia, which is distinguished either in low-grade or high-grade [4], and, eventually, to cancerization; in fact, it is estimated that BE enhances EAC risk by 30–125 fold [5].

It has been shown that BE harbors clonality features that resemble the ones we see in a EAC even if the morphology is not yet significant; this evidence supports the theory that BE corresponds to esophageal “field cancerization”, first described by Slaughter et al. in 1953 for oral cancer [6,7,8].

ESCC arises most frequently in the middle third of the organ, followed by the lower third and, lastly, the upper third; it is linked to chronic alcohol intake, tobacco use (chewing and/or smoking), hot beverage consumption, and genetic factors. Unlike EAC, in ESCC, esophageal epithelium progresses from low and then high-grade intraepithelial neoplasia (dysplasia) into cancer [9].

MicroRNA (miRNA) is a category of non-coding RNAs with a length of 18–22 nucleotides. The discovery of these peculiar molecules dates to 1993, when a 22-nucleotide long, non-coding RNA was found to post-transcriptionally regulate the expression of a gene in *C. elegans*. In the following years, hundreds of miRNAs have been detected and studied in humans, but their full potential is far from being entirely understood [10].

Like protein-coding messenger RNAs (mRNAs), miRNAs are transcribed by RNA polymerase II, undergo splicing, capping, and poly-adenylation and become so-called primitive miRNA or pri-miRNA. pri-miRNA transcripts are processed by Drosha, a RNase III endonuclease, to the hairpin precursor miRNAs (70 nucleotides), which is then transported into the cytoplasm. The precursor miRNA is then processed into 22 nucleotides miRNA duplexes by the RNase Dicer. Under a standard nomenclature system, the prefix “miR-” is followed by a dash and a number, the latter often indicating order of naming. A capitalized “miR-” refers to the mature form of the miRNA, while the uncapitalized “mir-” refers to the precursors (pre-miRNA or pre-miRNA). miRNAs with nearly identical sequences, except for one or two nucleotides, are distinguished with an additional lower-case letter. Moreover, two mature microRNAs originating from opposite arms of the same pre-miRNA are found in roughly similar amounts and are denoted with a -3p or -5p suffix [11].

miRNAs are ideal candidate biomarkers because are highly stable in tissues and body fluids. In plasma, microRNAs are included in RNA-binding multiprotein complexes and/or exosomes, which makes microRNAs less prone to degradation [12].

In most cases, miRNAs act by binding to the 3′-untranslated region (3′-UTR) of their target messenger RNAs (mRNAs), resulting in mRNA silencing or degradation, thereby interfering with protein translation. A single miRNA can bind and regulate multiple mRNAs; it is in fact estimated that miRNAs as a whole can modulate the translation of 50% of the protein codified by the mammalian genome [13,14]. 

The first clues of the role of miRNAs in cancer have been found in chronic lymphocytic leukemia, in which miR-15 and miR-16 were found to be down-regulated [15]. 

From that moment on, the role of miRNAs in cancer has been further researched, and its importance is presently recognized in many cancer types [16,17], such as thyroid cancer [18], lung cancer [19], renal cell carcinoma [20], breast cancer [21], endometrial cancer [22], gastric cancer [23], colorectal cancer [24], oral and oropharyngeal cancer [25], prostate cancer [26] and, of course, esophageal cancer and its precancerous conditions (i.e., BE and low- and high-grade intraepithelial neoplastic lesions) [27]. 

In this review, we aim to describe the main alterations in the expression of miRNAs in both EAC and ESCC, their significance in cancer development, their prognostic role, and some of the most promising future perspectives about the therapeutic possibilities concerning miRNAs.

## 2. Deregulated miRNAs in EC and Precancerous Lesions

miRNAs play a crucial role in the maintenance of cellular homeostasis by regulating several post-transcriptional pathways. In cancer, miRNAs are usually distinguished into two categories: oncomiRs, which are generally overexpressed, and tumor-suppressive (TS) miRNAs, which are down-regulated. One single miRNA can act as an oncomiR for a certain cancer type and as a TS miRNA for another, so that the two categories of miRNAs can partially overlap [28]. 

In this section, we will be focusing on the miRNAs that act as oncomiRs and TS miRNAs in esophageal cancer, their biological functions, and their prognostic implications. Table 1 summarizes the main miRNAs involved in esophageal carcinogenesis. 

### 2.1. miRNAs in Esophageal Precancerous Lesions

As previously mentioned, the two most common histotypes of EC have precancerous lesions, namely BE for EAC and intraepithelial squamous neoplasia for ESCC. Various studies tried to identify a molecular signature associated with these morphologic entities, to better comprehend the series of events that leads the transformation of normal epithelium into cancer. 

BE was found to harbor numerous specific alterations in miRNA expression when compared with healthy epithelium (HE) and EAC [85]. In fact, Wu et al. showed that the transition from HE to BE entails the highest number of differentially expressed miRNAs when compared with the following stages of progression, as the progressions from BE to BE with low- and high-grade dysplasia and from dysplastic BE to EAC [86]. The same study showed that the miRNA signatures in BE may anticipate other molecular features, since the burden of miRNA deregulation is quantitatively highest in this first phase, while the following steps are characterized by numerous DNA and mRNA alterations [86]. Nevertheless, substantial miRNA alterations have also been detected between BE and EAC; in fact, a study identified 27 miRNAs which were significantly up-regulated and 17 which were instead down-regulated in EAC, in comparison with BE [29]. Examples of miRNAs that were detected with higher incidence in EAC than in BE were miR-424, -663b, -502-5p, and -206. On the contrary, miR-215, -31-5p, -98 and -15b-5p were more often found in BE than in EAC [29]. Furthermore, this study looked for the miRNAs with the highest fold-changes between BE and EAC. Although three miRNAs were up-regulated by >10 fold in EAC, namely miR-4286, miR-630, and miR-575, only miRNA 205-5p was up-regulated by >10 fold in BE [29].

Another study investigated the role of the miR-200 family in esophageal carcinogenesis, whose members were found to be down-regulated in BE with high-grade dysplasia; furthermore, miR-200c was down-regulated in BE adjacent to EAC. These findings indicated that this family of miRNAs plays a role in the progression of the disease [87]. Moreover, the dysregulation of miR-125a-5p/125b has been found to be an early event in the esophageal (Barrett) oncogenesis and correlated inversely with HER2 status [31].

On the contrary, relatively few studies have focused on miRNA expression in esophageal squamous dysplasia; nevertheless, data have been collected to find a miRNA signature in esophageal squamous preneoplastic lesions. A study identified a set of seven circulating miRNAs (miR-16-5p, -197-5p, -320c, -451a, -486-5p, -638 and -92a-3p), which were found to be overexpressed in ESCC and squamous dysplasia when compared with controls. Among these seven, miR-197-5p, -320c, -638, and -92a-3p were also found to be overexpressed in dysplasia when compared with ESCC [30]; not only was this miRNA signature proposed to be a potentially useful biomarker of ESCC and dysplasia, but it also endorses the idea that miRNA variations can drive, or at least be associated with, the progression from normal epithelium towards cancerization.

### 2.2. Up-Regulated miRNAs in EC

The role of miR-675-3p has been investigated in esophageal cancer and in colorectal [88] and pancreatic cancer as well [89]. In ESCC, miR-675-3p has been found to be overexpressed when compared with healthy esophageal tissue. Its expression level has been positively linked to motility and invasiveness of cancer cells; furthermore, three possible targets of its modulatory activity have been proposed, namely E-cadherin and Metalloproteinases (MPPs) 2 and 9, which are involved in cancer cell motility, tumor progression, and metastasis. In particular, MMPs are responsible for the degradation of various extracellular matrix components [90], while E-cadherin is an adhesion molecule, and its loss is often linked to enhanced cell motility. Within ESCC, miR-675-3p levels have been found to be directly proportional to MMP2 and MPP9 expression. In summary, a decreased expression of E-cadherin and an enhanced expression of MPPs is linked with an improved migration ability and, consequently, an augmented invasiveness of the neoplasm [32]. 

miR-21 has been studied in many cancer types; it has been found to be dysregulated in breast cancer, gastric cancer and glioblastoma [91,92,93].

Within EC, the overexpression of miR-21 was linked with lymph node metastasis and with the depth of invasion of the neoplasm (T3 and T4 tumors showed higher concentration of miR-21 when compared with T1 and T2); furthermore, miR-21 was shown to be inversely related to the apoptotic rate of EC cells [33]. miR-21 performs its functions by interacting with the tumor suppressor gene *PTEN*, the gene Programmed Cell Death 4 (*PDCD4)* and the proto-oncogene *KRAS*. Moreover, a meta-analysis [34] showed that ECs which expressed higher miR-21 levels had a worse prognosis when compared with the ones with lower expression. Furthermore, in the early stages of ESCC carcinogenesis (namely squamous dysplasia (SD) and squamous intramucosal carcinoma (IM-SSC)) a correlation was found between the expression of PDCD4 and miR-21 [35]. PDCD4 is a well-established tumor suppressor and works as a protein translation inhibitor; its activity causes a decrease in tumor initiation, cell proliferation and cancer metastatic potential [94]. miR-21 was found to be inversely linked to PDCD4 expression in SD and IM-SSC, thus suggesting a potential role of PDCD4 as a biomarker for precancerous and early neoplastic condition in EC [35].

The role of miR-92a is well known in various malignancies, such as colorectal cancer [95] and chronic lymphocytic leukemia (CLL) B [96]. One of the putative roles of miR-92a in EC is to down-regulate E-cadherin expression; through this mechanism, EC cells gain motility, and they are more prone to lymph node metastasis [36]. Another study found a link between miR-92a and proliferation rate in EC; one of the causes of this occurrence seems to be the miR-92a-mediated down-regulation of PTEN. The same study found that higher expression of miR-92a was linked with a lower rate of apoptosis, possibly caused by the inhibition of the Bax/Bcl-2/Caspase-3 pathway [37]. Furthermore, a recent study found the up-regulation of circulating miR-92a in serum of patients with advanced precancerous lesions/early EAC in comparison with patients with non-neoplastic long-segment BE, demonstrating its possible role as mini-invasive diagnostic tool in secondary follow-up and management of BE patients [97]. 

miR-155 is among the most studied miRNAs and plays a role in numerous malignancies such as oral cancer [98], breast cancer [99] and non-small cell lung cancer [100]. In EC, miR-155 acts as an oncomiR; its putative function is to down-regulate the expression of Tumor Protein P53 Inducible Nuclear Protein 1 (TP53INP1) [38]. TP53INP1 is a pro-apoptotic protein whose expression is mediated by p53, which blocks the cell cycle in G1 [101]. Within EC, the down-regulated expression of TP53INP1 was found to be linked with an enhanced proliferation rate and this is thought to be caused by the impairment of the p53 pathway [38]. Another study found a link between the high concentration of miR-155 within EC and the lowered expression of MAP3K10 [39]. MAP3K10 is a serine/threonine kinase whose main activity is to activate the c-Jun N-terminal kinases (JNK; also known as MAPK8) signaling pathway, which is involved in pivotal cellular processes such as cellular differentiation and proliferation [102]. It was proposed that miR-155-mediated MAP3K10 down-regulation is responsible for chemo- and radio- resistance and an enhanced proliferation rate of EC [39].

miR-543 is a clear example of the oversimplification of the dichotomy between TS miRNAs and oncomiRs; in fact, it has a TS role in glioma, while it works as an oncomiR in renal cell carcinoma [103,104], other than in EC. *PLA2G4A* encodes for the Cytosolic Phospholipase A2 (cPLA2), which plays a crucial role in the regulation of the inflammatory pathways together with cyclooxygenase-2 (COX-2) and prostaglandin E synthase (PGES) [105]. Within EC, the expression of miR-543 was found to be inversely linked with PLA2G4A expression, which was found to be regulate the balance between E-cadherin and vimentin expression. The down-regulation of PLA2G4A favors the epithelial-mesenchymal transition (EMT) and consequently enhances the expression of vimentin (mesenchymal marker) and decreases the expression of E-cadherin (epithelial marker) [40]. Furthermore, another study found that the Long Intergenic Non-Protein-Coding RNA, P53 Induced Transcript (LINC-PINT), a miR-543 inhibitor, was able to decrease the proliferation rate of EC cells [41].

miR-27a was found to be up-regulated in EC and dysregulated in other malignancies as well, such as gastric [106] and colonic cancer [107]. In EC, miR-27 decreases the expression of F-Box and WD Repeat Domain-Containing 7 (FBXW7), which is a p53-dependent tumor suppressor [42]. The role of FBXW7 is to lead molecular targets towards ubiquitination and degradation through 26S proteasome [108]. Notch, NF-κB, Cyclin E and c-Myc are among its molecular targets [108]. Therefore, the down-regulation of FBXW7 in EC is thought to be linked to an enhanced proliferation rate [42].

The activity of miR-200a is well-recognized in various malignancies, such as bladder cancer [109] and endometrial cancer [110], and acts as an oncomiR in EC. The results of a study highlighted its involvement in the down-regulation of the Raf Kinase Inhibitory Protein (RKIP) [43]. RKIP has the pivotal function of regulating Raf-1 activity; in fact, RKIP binds Raf-1 preventing its activation, thereby down-regulating the MAP kinase (MAPK) pathway [111,112]. miR-200a activates the MAPK pathway via down-regulation of RKIP, thus promoting cancer invasiveness [43]. Furthermore, it has been shown that miR-200a promotes the expression of MMP14, which is then inhibited by RKIP. The expression of MMPs is known to enhance cancer mobility and invasiveness. The combination of these effects promotes EC invasive potential [43]. Another study demonstrated the involvement of miR-200a in the decreased expression of proteins such as PTEN, APC, and CDH1, among others [44]. PTEN is the main down-regulator of PI3K, which is part of a family of proteins involved in cell proliferation, migration and apoptosis and it is thought to act as a proto-oncogene [44,113]; Adenomatous Polyposis Coli Protein (*APC*) is a tumor suppressor gene that negatively regulates Wnt [114]; Cadherin 1 (CDH) is an important adhesion molecule. If lost, it is thought to cause enhanced mobility [115]. Furthermore, the expression of miR-200a was found to be linked with overall and disease-free survival in EAC [45].

miR-20b is another miRNA found to be overexpressed in EC and, as with miR-200a, can down-regulate the expression of PTEN, promoting proliferation, migration, invasion and inhibiting apoptosis in EC [46].

miR-371–373 cluster includes miR-371, miR-372, and miR-373, which are highly expressed in embryonic stem cells. The three members of this cluster are overexpressed in EC. It has been shown that this cluster is overexpressed in EC when compared with non-neoplastic adjacent tissue [47]. The putative target genes for this cluster of miRNAs are *TP53* and *LATS2* [47,48]. p53 is one of the most important molecules involved in the blockade of cell cycle and among the most studied proteins in cancer biology; LATS2 acts as a tumor suppressor by regulating the mitochondrial-mediated apoptosis [116]. Moreover, miR-373 appears to regulate the expression of Tissue Inhibitor of Metalloproteinases 3 (TIMP-3), which is an inhibitor of MMP; via TIMP-3 inhibition, miR-373 should enhance the migration ability and invasiveness of EC cells [48].

miR-9 is dysregulated in both non-neoplastic conditions, such as Alzheimer’s disease [117], and in neoplasms, acting as both an oncomiR and as a TS miRNA [118]. MiR-9 is up-regulated in EC and exerts an oncogenic function by down-regulating E-cadherin (CDH2) [49]. CDH2 is a calcium-dependent adhesion molecule, which plays a role in multiple malignancies, such as testicular germ cell tumors [119] and thyroid cancer [120] and is also a target of miR-675-3p, as previously mentioned. In EC, the reduced expression of CDH2 is thought to be linked with enhanced motility and metastatic potential of cancer cells. The putative mechanisms through which this can take place are mainly two: i) loss of cell-to-cell adhesion; ii) release of β-catenin and activation of its pro-mitotic target genes (such as *c-myc* and *cyclin D1*) [49]. Furthermore, high plasmatic levels of miR-9 were found to be significantly correlated with poor EC differentiation [50], while high levels of miR-9 within the neoplastic tissue were found to be linked with lymph node metastasis and poor survival rate [49].

miR-183 is a member of the miR-96-182-183 cluster, a well-studied group of miRNAs with the aforementioned ability to exert both an oncogenic and a TS function [121]. Within EC, miR-183 was found to be up-regulated. It acts by down-regulating the expression of PDCD4, thereby promoting cancer proliferation and decreasing the apoptotic rate [51]. Furthermore, miR-183 was found to interact with Forkhead Box Protein O1 (FOXO1) [52]. *FOXO1* is a tumor suppressor gene and its down-regulation via miR-183 has been observed in many cancer types [122]. Within EC, its down-regulation seems to enhance the drug-induced apoptotic rate [52]. A further mechanism has been suggested to explain the role of miR-183 in EC: the negative regulation of the NESH tumor suppressing pathway through the inhibition of ABI3BP [53]. Compelling evidence indicates that ABI3BP impairs the metastatic potential of cancer cells, lowers the apoptotic rate, hence its inhibition promotes the migration and invasive potential of cancer cells [123]. 

miR-223 is significantly up-regulated during Barrett carcinogenesis and has been documented as a potential circulating biomarker to discriminate patients with early EAC [54]. Data indicated that miR-223 exerts its effects by down-regulating FBXW7/hCdc4 expression at the post-transcriptional level and appears to regulate cellular apoptosis, proliferation, and invasion [55]. 

### 2.3. Donwregulated miRNAs in EC

miR-200b is among the most studied miRNAs in cancer [124,125]. In EC, miR-200 down-regulates the Wnt pathway by inhibiting the CDK2/PCNA-Associated Factor (PAF) axis [56,57]. 

In fact, PAF appears to be miR-200b most relevant target; PAF positively regulates the Wnt pathway, thereby promoting cancer cell growth [56], [57]. The down-regulation (or complete loss) of miR-200b within EC is linked to a dysregulation of the Wnt pathway, leading to an enhanced cell growth [57]. Furthermore, it has been found that miR-200b inhibits the expression of Kindlin-2, a protein that mediates various phases of carcinogenesis, resulting in the impairment of the AKT pathway. Therefore, the down-regulation of miR-200b leads to an uncontrolled activation of AKT, which is a frequent finding in EC [58].

miR-124 has a well-established role as a prognostic marker in many cancer types [126]. In EC, miR-124 was found to be down-regulated in comparison with normal adjacent tissue [59]. MiR-124 is involved in EC progression by regulating the expression of its target gene *NRP1* [59]. NRP1 is a receptor capable of binding many growth factors, such as TGF-β, PDGF and VEGF [127]. In EC, the down-regulation of miR-124 is responsible for NRP-1 overexpression [59] and is linked to poor prognosis, high clinical stage and cell migration [59]. Furthermore, miR-124 acts as a TS by regulating the expression of the pro-apoptotic protein Programmed Cell Death 6 (PDCD6) [60,128,129]. Furthermore, STAT3, which is an important regulator of proliferation and is overexpressed in EC, has be found to be another target of miR-24 [61,130,131].

miR-126 is a TS miRNA whose role has been evaluated in various neoplasms [132]. In EC, miR-126 appears to be down-regulated and inversely correlated with cell proliferation via modulation of Vascular Endothelial Growth Factor A (VEGF-A), which regulates proliferation and angiogenesis [62,133]. Moreover, miR-126 exerts its TS function by targeting PIK3R2 [63]. *PIK3R2* is an oncogene whose role is to activate the PI3K/AKT pathway, which is up-regulated in many malignancies [134]. MiR-126 has been reported to inhibits proliferation, colony formation and migration via down-regulation of PIK3R2 [63]. A disintegrin and metalloproteinase domain-containing protein 9 (ADAM9) expression, which promotes migration in cancer cells, was found to be inversely correlated with miR-126 [64].

miR-148a has been found to be dysregulated in many malignancies [135,136]. Within EC, miR-148a has the putative role of modulating the expression of Activin A Receptor Type 1 (ACVR1). ACVR1 is well-known cancer driver which is involved in SMAD signaling and belongs to the BMP/TGF-β receptor superfamily [137]. ACVR 1 is a direct target of miR-148a, with miR-148a being down-regulated and ACVR1 being overexpressed in ESCC. A study showed that the down-regulation of miR-148a, via modulation of ACVR1, was associated with enhanced expression of stem cell markers and increased proliferation rate of stem cells [136]. Another study found a further target for miR-148a, namely MAP3K, which activates the oncogenic ERK/MAPK pathway thereby promoting proliferation [66,138].

miR-26a plays an ambivalent role in various neoplasms, acting as both an oncomiR and a TS miRNA [139,140]. In ESCC it has a TS role by targeting cyclooxygenase 2 (COX-2) and has been found to be down-regulated [139]. COX-2 is an enzyme responsible for synthesis of prostaglandin and it is highly expressed in sites of inflammation and across many cancers as well [141]. In ESCC, the decreased expression of miR-26a was associated with a higher proliferation rate and greater migration ability of cancer cells [139]. Furthermore, miR-26a together with miR-26b were also found to directly target MYC Binding Protein (MYCBP), which binds and activates the oncogenic protein MYC [142]. In ESCC, the down-regulation of miR-26a and miR-26b leads to the overexpression of MYCBP and to an enhanced oncogenic activity of MYC related genes [68].

miR-199 family is also found to be deregulated in EC, in particular, miR-199a-3p and miR199a-5p are the ones of greatest interest.

MiR-199a-3p has been found to interact directly with Adenylate Kinase 4 (AKA4), lowering its expression. AK4 is a mitochondrial protein whose role is to promote survival in response to oxidative stress [143]. This interaction would result in an increased expression of AK4 in EC with low miR-199a-3p, which has been found to be possibly linked with radiotherapy resistance [69]. Of note, a similar effect has been found in osteosarcoma [144]. Furthermore, the down-regulation of miR-199a-3p plays a role in EC is via modulation of P21-Activated Kinase 4 (PAK4) expression [145]. PAK4 is thought to play a role in cancer as a target of K-Ras-driven malignancies, and it has been found to be overexpressed in many neoplasms [146].

MiR-199a-5p acts by modulating the expression of MAP3K11, which is a positive regulator of the JNK pathway [70]. In EC, the overexpression of MAP3K11, resulting from miR-199a-5p down-regulation, has been postulated to affect cancer cell proliferation [145]. 

miR-195 can regulate cancer cell proliferation and invasiveness by modulating the expression of its target Cell Division Cycle 42 (Cdc42), which is normally involved in the cytoskeleton formation and organization [71,147]. In ESCC, miR-195-Cdc42 axis has a prognostic role, with the down-regulation of miR-195 being linked to poor prognosis [147]. Furthermore, miR-195 seems to be involved in EC is through the modulation of Yes Associated Protein 1 (YAP1) expression. YAP1 is one of the most important effectors of the Hippo pathway and plays an important role in tumorigenesis and modulation of the tumor microenvironment [148]. YAP1 seems to be a direct target of miR-195; the overexpression of YAP1 caused by the down-regulation of miR-195 has been found to be linked to EC cells proliferation and apoptosis rate [73]. Another study found an inverse correlation between miR-195 and HMGA2, which is an oncogenic protein, and it is an important regulator of cell cycle and apoptosis via the activation of the PI3K/AKT pathway [74,149]. In EC, it was observed a down-regulation of miR-195, which is responsible for an enhanced proliferation rate of neoplastic cells through the overexpression of HMGA2 [74]. Of note, a similar mechanism has been found in lung cancer [149].

miR-27a can act both as a TS miRNA and as an oncomiR [75,150,151]. In EC, miR-27a performs its TS role by decreasing KRAS expression [75]. In ESCC the down-regulation of miR-27a causes an overexpression of the oncogene KRAS, enhancing proliferation and tumor progression [75].

miR-375 is a largely studied miRNA in cancer biology [152]. Metadherin (MTDH) is a protein which pays a crucial role in tumorigenesis, from initiation to metastasis, and in chemoresistance; MTDH interacts with the most important signaling pathways in cancer, such as PI3K/AKT, NF-κB, Wnt/β-catenin and MAPK [153]. MiR-375 was found to directly target MTDH, so that the down-regulation of this TS miRNA in EC results in the up-regulation of MTDH; this seems to be linked to cancer proliferation, invasion and cell cycle arrest [76]. Another study found a different mechanism responsible for the role of miR-375 in EC, which is the modulation of Specificity Protein 1 (SP1) expression [77]. SP1 is a transcription factor which has the function of regulating the expression of multiple genes involved in many cellular activities, such as proliferation, apoptosis, and differentiation; moreover, SP1 was found to be linked with the migration ability of some neoplasms [154]. In ESCC, miR-375 was found to be down-regulated as opposed to its target SP1; furthermore, the down-regulation of miR-375 was linked with poor prognosis, advanced cancer staging, cell proliferation and colony formation [77]. miR-375 was also reported to be a modulator of Matrix Metallopeptidase 13 (MMP13) expression [78]. MMP13 degrades several components of the extracellular matrix, such as fibronectin and various types of collagen and it is directly down-regulated by miR-375, promoting cancer cell aggressiveness and migration ability [78]. 

miR-133b is a well-known mRNA with a unique role in human cancer [155]. In EC, miR-133b activity is regulated by a long non-coding RNA, namely lncRNA-TTN-AS1. lncRNA-TTN-AS1 promote expression of Snail1 and FSCN1 by competitively binding miR-133b, stimulating EMT cascade. Moreover, lncRNA-TTN-AS1 also induces FSCN1 expression by sponging miR-133b and the up-regulation of mRNA-stabilizing protein HuR, which further promotes ESCC invasion cascades [79]. The findings of another study indicated that miR-133b acts as a TS miRNA in EC, by modulating Epidermal Growth Factor Receptor (EGFR) expression [80]. In EC, miR-133b was found to inhibit migration and proliferation through the down-regulation of EGFR expression, so, the down-regulation of miR-133b should result in an enhanced EGFR expression [80].

miR-143 is another TS miRNA found to be down-regulated in EC. Multiple mechanisms have been postulated to explain its role. LIM And SH3 Protein 1 (LASP1) is involved in a wide range of cellular functions and its overexpression has been detected in many malignancies [81,156], among which EC [157]. The down-regulation of miR-143 has been found to increase the expression of LASP1, which enhances cell proliferation, migration, and invasion [81]. miR-143 also regulates the expression of Protein Quaking 5 (QKI-5). QKI proteins are involved in pre-mRNA splicing, mRNA transport and translation, and mRNA and miRNA stability [158]. Although QKI-5 acts as a tumor suppressor in many cancer types [158,159], within EC its expression has been found to be up-regulated and it was directly linked to the metastatic potential [81,160]; furthermore, the silencing of QKI-5 expression can increase apoptotic rate and decrease proliferation. A further study found that in EC miR-143 is inhibited via sponging by the long non-coding RNA HOTAIR (for HOX transcript antisense RNA) [82]. The same study found Hexokinase 2 (HK2) to be the putative target of miR-143. The down-regulation of miR-143 determined by HOTAIR results in the overexpression of HK2, which promotes cell proliferation, invasion and migration [82].

miR-125b has been studied in various malignancies, such as gallbladder cancer [161], gastric cancer [162] and breast cancer [163]; within EC, it was found to act as a TS miRNA. High Mobility Group AT-Hook 2 (HMGA2) is a protein with a structural role in replication forks, and it is expressed in cancer cells of a variety of neoplasms and in stem cells as well, while it is virtually absent in healthy adult cells; furthermore, it was successfully linked with poor prognosis in many cancer types [164]. The down-regulation of miR-125b, in EC, was associated with the overexpression of HMGA2, which is responsible for an enhanced proliferation rate, invasiveness and migration ability of cancer cells [83]. MiR-125b also regulates HCLS1 Associated Protein X-1 (HAX-1) expression, which is involved in mitochondria-dependent apoptosis. It was observed that the ectopic expression of HAX-1 in colon cancer has an antiapoptotic effect on cancer cells [165]. In EC, the reduced expression of miR-125b should thus result in the up-regulation of HAX-1 with a putative positive effect on cell proliferation and migration [84]. Table 2 summarizes the oncomiRs and TS miRNAs found to be dysregulated in EC and their putative targets.

## 3. Future Perspectives: Liquid Biopsy, Drug Resistance, and miRNA-Based Therapies

Since their discovery, miRNAs have been under continuous investigation to better define their role in various biological mechanisms.

As previously mentioned, miRNAs are thought to play a significant role in all the phases of cancer development, from initiation to metastasis; for this reason, miRNAs represent promising prognostic and predictive biomarkers in cancer and they also have therapeutic potential [166]. As diagnostic tools, miRNAs have been proved to be detectable in formalin-fixed paraffin-embedded (FFPE) specimens, in fine needle aspiration biopsies (FNABs) and in various body fluids, such as plasma, saliva, and urine [167,168]. In this context, it was shown that the level of miR-34a-5p increased, while miR-148a-3p and miR-181a-5p decreased in blood samples of patients with EC when compared with controls; moreover, miR-181a-5p was proposed as a biomarker for early EC [169]. MiR-21 and miR-375 also were tested for their diagnostic potential in EC in two different studies; miR-21 was found to be up-regulated in serum samples from patients with EC, while miR-375 was found to be down-regulated. Therefore, a high miR-21/miR-375 ratio was proposed as a diagnostic biomarker for early EC [91,170]. Another study found that miR-375 together with miR-100 were also down-regulated in blood samples from EC patients; on the contrary, the same study found a significant higher expression of miR-25 and miR-151, thus proposing another conceivable diagnostic panel [171]. 

It is widely acknowledged that miRNAs act as mediators of anti-cancer drug sensitivity [169], so it is not surprising that many microRNAs have been proposed as biomarkers for response to therapy in various malignancies, among which EC is no exception. Table 3 outlines some examples of microRNAs whose expression was linked with response to various anti-cancer therapies.

Playing such an important role in esophageal carcinogenesis, miRNAs have been proposed and tested as a therapeutic option [179]; however, some authors sustain that many pharmaceutical companies have lost interest in developing RNA-based therapies [180]. Conceptually, miRNA-based therapy is focused either on reducing the activity of onco-miRNAs (through “miRNA inhibitors”) or to enhance the activity of TS miRNAs (through “miRNA mimics”) [181]. With the purpose to deliver a miRNA-based drug into a tumor, two choices are possible, namely local and systemic administration; of course, each of these options have advantages and downsides: for example, through local administration, it would be easier to reach therapeutic concentrations within the neoplasia, while systemic administration has the benefit of reaching the primary tumor and possible secondary localizations [179]. Unfortunately, there are a few important limitations: miRNA mimics have shown to be ineffective at low doses and to cause non-specific changes in gene expression when administered at high dosage; miRNA inhibitors, on the other hand, have been shown to produce cross-reactions among miRNAs that belong to the same family [181]. Many of the studies that were mentioned in chapter 2 used miRNA mimics or miRNA depletion systems (such as miRNA sponges) to evaluate the consequences of the manipulation of these molecules with the purpose of testing their hypotheses in vitro or in xenograft models, nevertheless. However, to the best of our knowledge, miRNA-based therapy for EC and for any other cancer has not reached the clinic yet [182].

## 4. Conclusions

EC is among the most common gastrointestinal neoplasms; however, it is still burdened by a poor prognosis. MiRNAs are post-transcriptional regulators and have been proved to modulate several oncogenic and tumor-suppressive pathways involved in cancer development. Numerous studies have found that miRNAs are involved in all the phases of carcinogenesis, from initiation to metastatic spread. 

In this work we give an insight on the dysregulation of various miRNAs and miRNA signatures in esophageal preneoplastic lesions. Interestingly, miRNAs seem to be progressively dysregulated during the carcinogenetic cascade and the transition from HE to BE entails the highest number of differentially expressed miRNAs. Furthermore, we give an overview of the dysregulation of oncomiRs and TS miRNAs in EC and of the molecular mechanisms that underlie their functions. 

Considering their role in cancer development and because miRNAs can be detected rapidly and efficiently in tissues and body fluids, they are ideal candidate biomarkers for diagnostic purposes and prognostic and predictive stratification of patients [183]. Furthermore, new insights into the role of miRNAs in cancer biology, have made miRNAs attractive tools and targets for novel therapeutic approaches. However, while the use of miRNA mimics and inhibitors is currently under investigation, it has not reached the clinic yet.

In our opinion, one of the most important limitations of some of the miRNA-related studies that were summarized in this paper is that they are based on in vitro techniques, and they are not able to reproduce the complexity of the primitive cancer environment and the pathophysiology of the host. Moreover, the artificial overexpression and/or knockdown of the miRNAs do not necessarily represent a correct way to evaluate the biological behavior these molecules; thus, the results obtained by concentration measurement do not represent a completely accurate surrogate of the post-transcriptional regulation of gene expression [184]. In addition, there is evidence that only very high concentrations of miRNAs can actually perform a significant target gene down-regulation. Even highly expressed miRNAs, in some cases, do not modify the expression of their targets; this is thought to be due to the nuclear localization of some miRNAs or to their configuration in an inactive state [185]. 

In conclusion, compelling evidence points out the complexity of the mechanisms involved in post-transcriptional regulation performed by miRNAs. Nevertheless, further research is needed to fully comprehend their role in EC carcinogenesis and to translate this knowledge into clinical applications.

## Figures and Tables

**Table 1:** Principal up-regulated (oncomiRs) and down-regulated (TS miRNAs) dysregulated in esophageal precancerous lesions and EC.

**Precancerous Lesions**	Up-regulation of miR-215 [29], miR-31-5p [29], miR-98 [29], miR-15b-5p [29], miR-197-5p [30], miR-320c [30], miR-638 [30] and miR-92a-3p [30]
	Down-regulation of miR-424 [29], miR-663b [29], miR-502-5p [29], miR-206 [29], miR-200 family [29], miR125a-5p and miR-125b [31]
**Esophageal Carcinoma**	Up-regulation of miR-675-3p [32], miR-21 [33,34,35], miR-92a [36,37], miR-155 [38,39], miR-543 [40,41], miR-27a [42], miR-200a [43,44,45], miR-20b [46], miR-371-373 [47,48], miR-9 [49,50], miR-183 [51,52,53], miR-223 [54,55]
	Down-regulation of miR-200b [56,57,58], miR-124 [59,60,61], miR-126 [62,63,64], miR-148a [65,66], miR-26a [67,68], miR-199 family [69,70], miR-195 [71,72,73,74], miR-27a, [75], miR-375 [76,77,78], miR-133b [79,80], miR-143 [81,82], miR-125b [83,84]

**Table 2:** OncomiRs and TS miRNAs dysregulated in EC, their putative targets and the biological materials evaluated in the studies.

miRNA	Role in EC	Putative Target(s)	Evaluated Material(s)
miR-675-3p	OncomiR	E-cadherin, MMP2, MMP9	PS
miR-21	OncomiR	PTEN, PDCD4, KRAS	PS
miR-92a	OncomiR	PTEN, Bax/Bcl-2/caspase-3 axis;	CL (normal and cancerous)
		E-cadherin	PS
miR-155	OncomiR	TP53INP1	PS, CL
		MAP3K10	CL (cancerous)
miR-543	OncomiR	cPLA2	PS, CL
miR-27a	OncomiR	FBXW7	PS
miR-200a	OncomiR	RKIP, PTEN, APC, E-cadherin	CL (cancerous)
miR-20b	OncomiR	PTEN	PS, CL
miR-371-373 cluster	OncomiR	p53, LATS2	PS
miR-9	OncomiR	E-cadherin	PS, CL
miR-183	OncomiR	PDCD4	PS, CL
		FOXO1	PS, CL
		ABI3BP	CL (normal and cancerous)
miR-223	OncomiR	FBXW7/hCdc4 axis	PS
miR-200b	TS miRNA	CDK2/PAF axis	CL (cancerous)
		Kindlin-2	PS
miR-124	TS miRNA	NRP1, PDCD6, STAT3	PS, CL
miR-126	TS miRNA	VEGF-A	CL, XE
		PIK3R2, ADAM9	PS, CL
miR-148a	TS miRNA	ACVR1, MAP3K	PS, CL
miR-26a	TS miRNA	COX-2	PS, CL, XE
		MYCBP	PS, CL
miR-199 family	TS miRNA	AK4, MAP3K11	CL
		PAK4	PS, CL
miR-195	TS miRNA	Cdc42, HMGA2	PS, CL
		YAP1	CL (normal and cancerous)
miR-27a	TS miRNA	KRAS	CL (normal and cancerous), XE
miR-375	TS miRNA	MTDH, SP1, MMP13	TS, CL
miR-133b	TS miRNA	FSCN, Snail1	PS, CL, XE
		EGFR	PS, CL
miR-143	TS miRNA	LASP1, QKI-5	PS, CL
		HK2	PS, CL, XE
miR-125b	TS miRNA	HMGA2	CL (cancerous)
		HAX-1	PS, CL

Abbreviations: TS = tumor suppressive; EC = esophageal cancer; Patient Samples (cancer and paired normal esophageal tissue) = PS; Cell Lines = CL; Xenograft = XE.

**Table 3:** microRNAs involved in mechanism of anti-cancer therapy resistance.

Type of Resistance	microRNA
5-fluorouracil resistance	Down-regulation of miR-29 [172]
Platinum resistance	Down-regulation of miR-149, miR-218, miR-187 [173,174,175]
Radio-resistance	Up-regulation of miR-124, miR-199a-3p [142,176]
Chemoresistance	Up-regulation of miR-455-3p [177]
Radiochemo-resistance	Dysregulation of miR-21 and miR-93 [178]

## Data Availability

Data is available upon request.
